# Silymarin-Loaded Nanoparticles Based on Stearic Acid-Modified *Bletilla striata* Polysaccharide for Hepatic Targeting

**DOI:** 10.3390/molecules21030265

**Published:** 2016-02-29

**Authors:** Yanni Ma, Shaolong He, Xueqin Ma, Tongtong Hong, Zhifang Li, Kinam Park, Wenping Wang

**Affiliations:** 1 Institute of Clinical Pharmacology, General Hospital of Ningxia Medical University, Yinchuan, Ningxia 750004, China; yannima2015@sina.com; 2 Department of Pharmaceutics, School of Pharmacy, Ningxia Medical University, 1160 Shengli Street, Yinchuan, Ningxia 750004, China; 15296907978@163.com (S.H.); xueqinma2015@126.com (X.M.); lizhifang2015@sina.com (Z.L.); 3 Department of Pharmacy, General Hospital of Yankuang Group, Zou Cheng, Shandong 273500, China; tongtonghong2015@sina.com; 4 Departments of Biomedical Engineering and Pharmaceutics, Purdue University, West Lafayette, IN 47907, USA; kpark@purdue.edu

**Keywords:** silymarin, *Bletilla striata*, polysaccharide, nanoparticles, hepatic targeting

## Abstract

Silymarin has been widely used as a hepatoprotective drug in the treatment of various liver diseases, yet its effectiveness is affected by its poor water solubility and low bioavailability after oral administration, and there is a need for the development of intravenous products, especially for liver-targeting purposes. In this study, silymarin was encapsulated in self-assembled nanoparticles of *Bletilla striata* polysaccharide (BSP) conjugates modified with stearic acid and the physicochemical properties of the obtained nanoparticles were characterized. The silymarin-loaded micelles appeared as spherical particles with a mean diameter of 200 nm under TEM. The encapsulation of drug molecules was confirmed by DSC thermograms and XRD diffractograms, respectively. The nanoparticles exhibited a sustained-release profile for nearly 1 week with no obvious initial burst. Compared to drug solutions, the drug-loaded nanoparticles showed a lower viability and higher uptake intensity on HepG2 cell lines. After intravenous administration of nanoparticle formulation for 30 min to mice, the liver became the most significant organ enriched with the fluorescent probe. These results suggest that BSP derivative nanoparticles possess hepatic targeting capability and are promising nanocarriers for delivering silymarin to the liver.

## 1. Introduction

Silymarin (SM) is a flavonolignan mixture derived from the seeds of *Silybum* *marianum* L. Gaertn (milk thistle), with silybin as its main component (70%–80%) [1]. SM has been widely used as a hepatoprotective agent for a variety of acute and chronic liver diseases, due to its therapeutic effect based on its antioxidant [2], anti-inflammatory [3], immunomodulatory [4] and anti-viral activities [5]. Previous reports have shown that the efficacy of oral SM on liver function and chronic hepatitis C virus (HCV) load was low, due to its low bioavailability, extensive first pass metabolism and short life of flavonoids [6]. However, intravenous (i.v.) SM was well tolerated and exhibited a substantial antiviral effect against in non-responders [7]. Thus, introducing SM as a safe and efficient i.v. formulation is a major challenge. A few attempts have been made to explore the parenteral administration of SM. Christodoulou *et al.* developed a water-soluble silibinin-hydroxypropyl-β-cyclodextrin lyophilized product, but drug level rapidly decreased in serum and drug distribution in heart, kidney and liver was similar after i.v. administration (20 mg/kg) to mice [8]. In the work of Yliperttula’s group, β-sitosterol β-d-glucoside (Sito-G) was added to the liposome formulation to enhance the uptake of SM in HepG2 cells, yet the *in vivo* performance was not determined [9]. Therefore, further studies on novel drug delivery system are required for liver-targeting and prolonged therapeutic effect.

Amphiphilic polymers can associate into nanoscaled interpolymeric self-aggregates with a hydrophobic core and a hydrophilic shell in aqueous media. Among these polymers, hydro-phobically-modified polysaccharides have attracted much attention due to their biodegradability and biocompatibility, and their potential application as drug vectors has been widely explored, especially for delivery of poorly soluble drugs [10,11,12]. Moreover, self-assembled nanoparticles with targeting ligands are promising carriers for anti-cancer drugs, which are aimed are providing enhanced therapeutic efficacy and reduced systematic toxicity [13]. However, the conjugation of polymers with targeting agents usually involves a complex procedure and induces a decreased yield of derivatives. Sometimes polysaccharides themselves can act as active agents due to their bioactivity, and hyaluronic acid is a typical example [14]. Most commonly used polysaccharides include pullulan, chitosan, cellulose, dextran, heparin and hyaluronan [15]. Other natural polysaccharides are seldom investigated to develop micelle systems.

*Bletilla* *striata* polysaccharide (BSP), as an extract from the tubers of *Bletilla* *striata*, is a neutral water-soluble glucomannan with a backbone of (1 → 4)-linked β-d-mannose and glucose in a molar ratio of 3:1. In our previous study [16], fatty acids with different chain lengths were used to modify BSP, and the BSP hydrophobically modified by stearic acid (hm-BSP) showed a preferable self-assembly property, as well as, good biocompatibility.

The aim of the present work was to evaluate the potential application of the stearic acid modified-BSP micelles for liver delivery of SM. SM-loaded nanoparticles based on hm-BSP were prepared and their morphology, particle size, thermal and crystalline properties characterized, and their *in vitro* release studied as well. The HepG2 cell line was applied to evaluate the cytotoxicity and cell uptake of the obtained nanoparticles, and biodistribution in mice after intravenous administration of nanoparticle and solution formulations was also observed under an imaging system.

## 2. Results and Discussion

### 2.1. Preparation and Characterization of Silymarin-Loaded Nanoparticles

**Figure 1:**
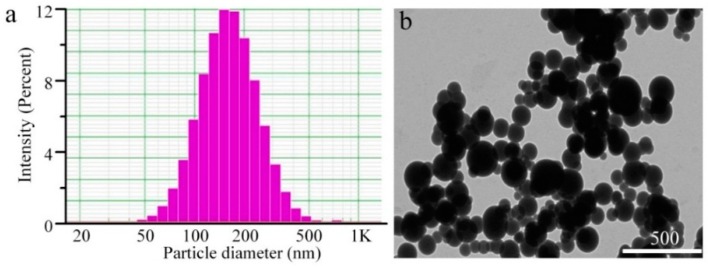
(**a**) Particle size distribution and (**b**) TEM image of SM-hm-BSP nanoparticles.

Figure 1 shows the size distribution and morphology of drug-loaded nanoparticles by DLS and TEM, respectively. SM-hm-BSP nanoparticles were almost spherical in shape under TEM. Their particle size was around 180 nm when observed under TEM and 200 nm as determined by DLS, which is mainly due to the different preparation processes of the samples. TEM depicts the size of the samples in a dried state, whereas, DLS depicts the size in the hydrated state. 

According to our previous work, the average size of empty nanoparticles was 250 nm [16], which was smaller than that of the drug-loaded nanoparticles. The reason might be the encapsulation of a hydrophobic drug leads to a more compact core-shell structure.

Table 1 summarizes the characteristics of the SM-hm-BSP nanoparticles. The zeta potential of the nanoparticles was nearly neutral in water, and the EE of silymarin-loaded nanoparticles was approximately 80% at a theoretical DL of 10%.

**Figure 2:**
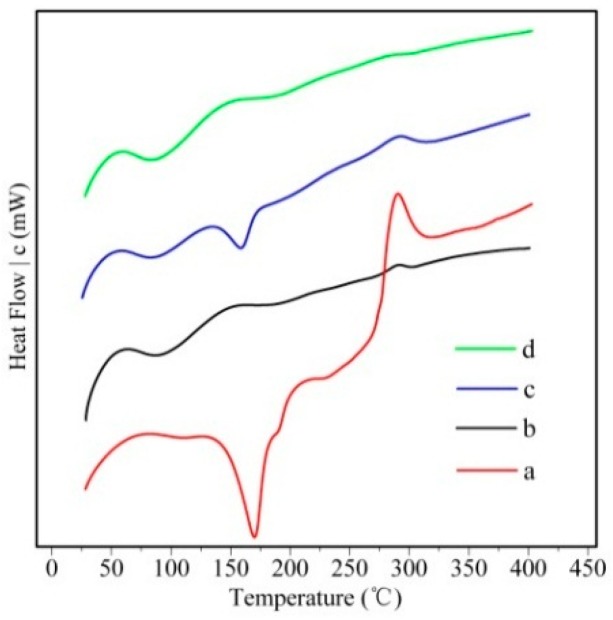
DSC thermograms of (**a**) SM; (**b**) hm-BSP; (**c**) physical mixture and (**d**) SM-hm-BSP nanoparticles.

DSC thermograms of drug, polymer, physical mixture and drug-loaded nanoparticles are illustrated in Figure 2. The polymer showed a wide endothermic peak at 80 °C. SM powder showed a sharp endothermic peak at about 165 °C corresponding to its melting point and then decomposed above 270 °C, which was also observed by Jia *et al.* [17]. The peaks of drug and polymer were retained in the curve of their physical mixture. In contrast, no melting peak corresponding to the fusion of SM was observed in the curve of drug-loaded nanoparticles, indicating a loss of crystallinity after encapsulation in the hm-BSP nanocarriers.

**Figure 3:**
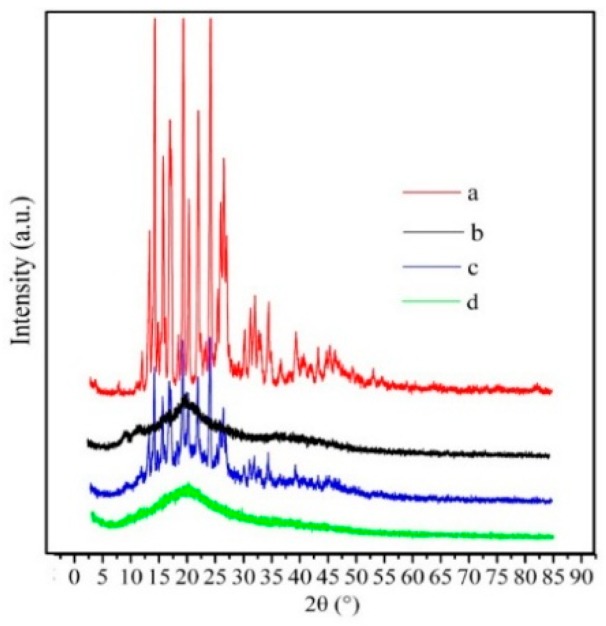
XRD patterns of (**a**) SM; (**b**) hm-BSP; (**c**) physical mixture and (**d**) SM-hm-BSP nanoparticles.

To further confirm that SM was successfully loaded into the hm-BSP nanoparticles, XRD patterns of drug, polymer, the physical mixture and drug-loaded nanoparticles were recorded and are shown in Figure 3.

It can be observed that the XRD spectra show broad peaks in the hm-BSP. The major peaks of crystalline SM (at 2θ range of 12° to 35°) were detected in diffractograms of SM powder [18], and the physical mixture as well. However, the specific peaks of SM crystals disappeared in the drug-loaded nanoparticles, indicating that SM was molecularly dispersed within the nanocarriers. These results indicate that SM was successfully encapsulated into the nanoparticles as a molecular dispersion [19].

### 2.2. In Vitro Release Study

**Figure 4:**
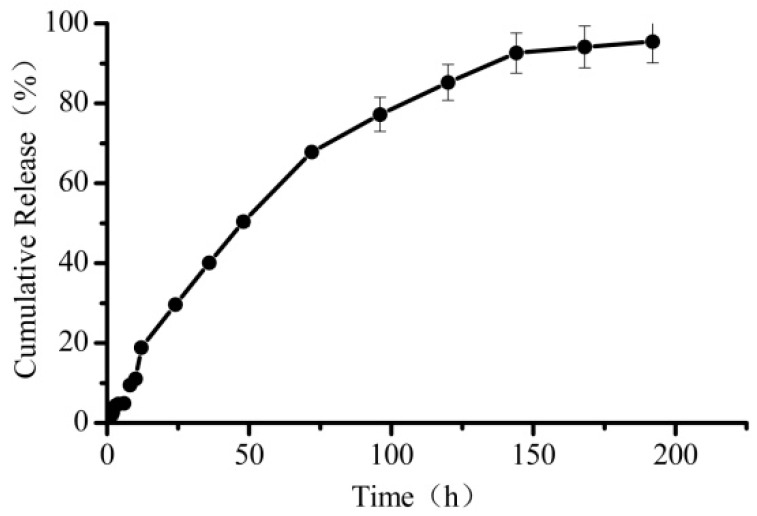
*In vitro* drug release profiles from SM-hm-BSP nanoparticles in pH 7.4 PBS.

The *in vitro* drug release behavior of SM-hm-BSP nanoparticles was determined in pH 7.4 PBS at 37 ± 0.5 °C. As shown in Figure 4, the SM-hm-BSP nanoparticles showed a sustained release pattern with no obvious initial burst release. It follows a typical drug release from a matrix formulation, in which the drug release in the beginning is higher than that of later times. The steady release state lasted for almost one week and up to 95% of the drug was released by the end of the experiment. The slow drug release may have resulted from the electrostatic interaction between the drug and self-assembled micelles [20]. The week-long release of silymarin is interesting for a few reasons. First, it is not known whether the *in vitro* drug release is reproduced *in vivo*. Most i.v. administered drug delivery systems have not been examined for their *in vivo* drug release properties. Assuming the week-long release *in vivo*, however, it presents another related question. Since it is known that nanoparticles will not circulate in the blood for a week, the usefulness of the week-long drug release remains to be seen. It is possible, however, that the nanoparticles accumulated in the liver may slowly release the drug over a period of a week, and this may present a new way of drug delivery to the target for effectiveness lasting a week or more.

### 2.3. Cytotoxicity Test

**Figure 5:**
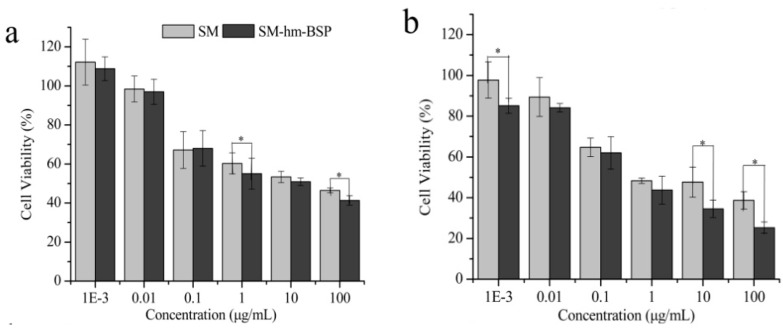

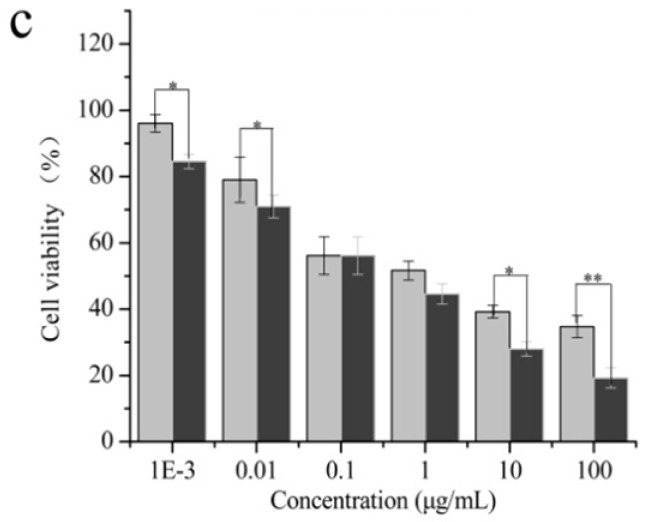
Cell viability of HepG2 cells treated with various concentration of SM and SM-hm-BSP nanoparticles for (**a**) 24 h; (**b**) 48 h and (**c**) 72 h. (*n* = 6). * *p* < 0.05; ** *p* < 0.01.

The cytotoxicity of SM and SM-hm-BSP on HepG2 cells at different intervals was evaluated through the CCK-8 assay. As shown in Figure 5, the inhibitory effect of free SM and the SM-hm-BSP nanoparticles on cell proliferation increased over a time period of 72 h. and the cell suppression of free drug and drug-loaded nanoparticles against HepG2 cell lines exhibited a dose-dependent effect. Compared to SM solution (IC_50_ = 7.50 μg/mL), SM-hm-BSP nanoparticles exhibited enhanced growth inhibition effects on HepG2 cells in almost all concentrations in 72 h, and showed a significantly lower IC_50_ value (0.66 μg/mL). These findings indicate that the pharmacological activity of SM was enhanced after encapsulated into hm-BSP nanoparticles.

It is interesting that SM-hm-BSP nanoparticles had much better anti-cancer cell proliferation activity than free SM itself. Although drug release from the nanoparticles was slow. SM-hm-BSP nanoparticles showed a significant inhibition effect on HepG2 cell proliferation compared to the SM solution. Since hm-BSP itself was proven to be a preferable cytocompatibility material [16], we assumed that the main reason for the difference in cytotoxicity was that the micelles had a high affinity for hepatocytes. To confirm this hypothesis, the cellular internalization capacity of HepG2 cells for free C6 and C6-hm-BSP nanoparticles were further investigated by fluorescence microscopy and fluorometry, respectively.

### 2.4. Cell Uptake and Flow Cytometry Study

**Figure 6:**
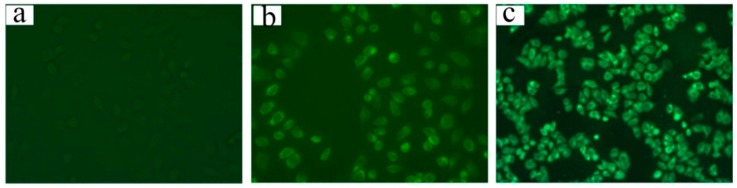
Fluorescence images (×200) of HepG2 cells treated with (**a**) medium; (**b**) free C6 and (**c**) C6-loaded nanoparticles for 1 h. The C6 content in micelle solutions was 0.089 μg/mL.

Figure 6 shows the fluorescence microscopy images of HepG2 cells following incubation with the free C6 and C6-hm-BSP nanoparticles for 1 h. Cells incubated with the medium (control) showed almost no fluorescence (Figure 6a), and those with the free C6 solution had a weak fluorescence (Figure 6b). In contrast, a strong fluorescence was observed in the cells after incubation with the C6-hm-BSP nanoparticles (Figure 6c), indicating that C6 in C6-hm-BSP nanoparticles had an improved cellular uptake.

**Figure 7:**
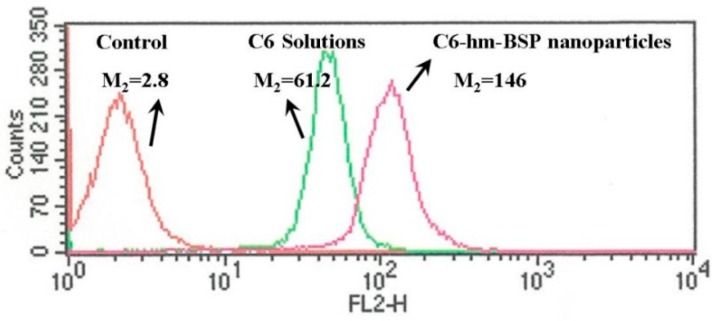
Fluorescence intensities of HepG2 cells treated with medium, free C6 and C6-loaded nanoparticles for 1 h. The C6 content in micelle solutions was 0.089 μg/mL.

The mean fluorescence intensity of the cells treated with the free C6 and C6-hm-BSP nanoparticles was quantitatively analyzed by a flow cytofluorometer. As shown in Figure 7, the concentration of C6 in HepG2 cells treated with C6-hm-BSP nanoparticles was 2.4-fold higher than that in cells incubated with free C6. Thus, hm-BSP nanoparticles achieved an excellent drug delivery effect, which is in agreement with the earlier results that fatty acid modified glucomannans have the potential to act as gene delivery vectors [21].

### 2.5. Tissue Distribution Study

**Figure 8:**
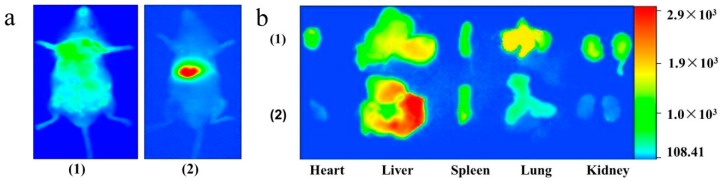
Fluorescence images of (**a**) mice body; (**b**) major organs of DIR after i.v. administration of DIR Solution **(1)** and DIR-labeled nanoparticles **(2)** into mice for 30 min.

As shown in Figure 8a, a nonspecific distribution of a fluorescent signal all over the body was observed after treatment with DIR solution for 30 min. In contrast, a strong signal was shown only in the liver region of the live mice after injection with DIR-loaded nanoparticles, suggesting that the fluorescence probe was mostly accumulated in the liver. The major organs were then removed and analyzed directly under the imager (Figure 8b). A decreased fluorescence intensity for DIR-loaded nanoparticles was observed in the lung, heart and kidney, in comparison with that for DIR solution. However, the fluorescence signal of DIR nanoparticles (2261.4) in the liver was 17.2-fold higher than that of free DIR (131.4), indicating that hm-BSP nanocarriers could selectively deliver more DIR molecules into the liver.

Taken together, the above results suggest that the hm-BSP nanoparticles will be promising liver-targeted delivery vectors, especially for hepatoprotective drugs with poor aqueous solubility. On the other hand, it also provides a reference for the development and application of functionalized polymers based on natural polysaccharides.

## 3. Experimental Section

### 3.1. Materials

Silymarin (≥98%) and coumarin 6(C6) were purchased from Sigma-Aldrich (St. Louis, MO, USA). 1,1′-Dioctadecyl-3,3,3′,3′-tetramethylindotricarbocyanine iodide (DIR) was bought from Amyjet Scientific Inc. (Wuhan, China). *N*,*N*′-Dicyclohexylvcarbodiimide (DCC) was acquired from Bioduly (Nanjing, China) and dimethylaminopyridine (DMAP) was from Kelong Chemical Regent Co., Ltd. (Chengdu, China). Phosphoric acid was obtained from Chemical Company (Tianjin, China). Polyethylene glycol (PEG400) was purchased from Shanghai Chemical Reagent Co., Ltd. (Shanghai, China). Dialysis tubing (molecular weight cut-off 3.4 kD) was supplied by Greenbird Biological Technology Co., Ltd. (Shanghai, China). Dulbecco’s Modified Eagle’s Medium (DMEM) and fetal calf serum (FCS) were obtained from Thermo Fisher Scientific, Inc. (Waltham, MA, USA). The tetrazolium-8-[2-(2-methoxy-4-nitrophenyl)-3-(4-nitrophenyl)-5-(2,4-disulfophenyl)-2*H*-tetrazolium] monosodium salt (CCK-8) was purchased from Dojindo Laboratories (Kumamoto, Japan). Methanol and acetonitrile were of HPLC grade. Phosphate-buffered saline (PBS; 0.01 M, pH 7.2–7.6) and other reagents were of analytical grade and used as received. Double distilled water was prepared in our laboratory.

### 3.2. Synthesis of hm-BSP

The hm-BSP derivative was synthesized according to the method reported by Sallustio *et al.* [22]. Briefly, BSP (0.100 g) was dissolved in DMSO (20 mL). Stearic acid (0.284 g) was activated by the addition of DCC (0.206 g) and DMAP (0.146 g) and dropped into BSP solution while stirring. The mixture was continuously stirred for 2 h at 80 °C, and then for 24 h at room temperature. The resulting solution was further dialyzed (molecular weight cut-off 3.4 kD) against water and freeze-dried to obtain the final product of hm-BSP.

### 3.3. Preparation of Silymarin-Loaded Nanoparticles

SM-loaded nanoparticles were prepared by an ultrasonication dispersion method [23,24]. Briefly, hm-BSP (25.0 mg) was dispersed in water at a concentration of 2.5 mg/mL, and then slowly treated dropwise with 0.5 mL drug solution in ethanol (5.0 mg/mL) followed by stirring at room temperature for 24 h. Subsequently, the mixture was treated by a probe-type Ultrasonic Processor (20–25 kHz, Ningbo Scientz Biotechnology Co. Ltd., Ningbo, China) at 400 W for 10 min in an ice-water bath. The period of ultrasound burst was set to 2 s with a pause of 3 s between two ultrasound bursts. The resultant solution was centrifuged at 3718× *g* for 20 min to remove the free drug. Finally, the supernatant was filtered through a 0.45 μm syringe filter to obtain the solution of drug-loaded nanoparticles. In addition, the nanoparticles loaded with C6 or DIR were also prepared by the same procedure for further study.

### 3.4. Characterization of Silymarin-Loaded Nanoparticles

To observe the morphology of drug-loaded nanoparticles, one drop of sample was deposited on the carbon-coated 300 mesh copper grid, air-dried and imaged using a JEM-100C transmission electron microscope (TEM, JEOL, Tokyo, Japan). Particle size and zeta potential measurements were carried out on a Nicomp 380 ZLS analyzer (PSS Nicomp, Santa Barbara, CA, USA).

Differential scanning calorimetry (DSC) was carried out using a SETSYS-1750 CS Evolution thermogravimetric analyzer (Setaram, Caluire-et-Cuire, France). Heating curves were recorded at a scan rate of 10 °C /min from 25 to 450 °C under a dry nitrogen atmosphere.

The crystalline state of SM, hm-BSP, physical mixture and drug-loaded nanoparticles were measured by an X-ray powder diffraction (XRPD) instrument (D/MARX2200/PC, Rigaku Co., Tokyo, Japan) using CuKα radiation at 40 mA and 40 kV. Standard runs were performed with a scanning rate of 0.02°/min over a 2θ range of 3–85°.

The drug content was analyzed by a HPLC (Model-L2000, Hitachi, Tokyo, Japan) method [25,26]. The analytical column was a Phenomenex C18 (5 μm, 4.6 × 150 mm). The mobile phase was composed of methanol, acetonitrile and water (16:34:50, *v*/*v*) and the final pH was adjusted to 4.0 with phosphoric acid, the flow rate was set at 0.8 mL/min and the column temperature at 35 °C. The detection was performed at 226 nm using an UV-VIS detector (Model: Hitachi L7420).

For determination of drug content inside the nanoparticles, 200 μL of sample was mixed with 10 mL DMSO and sonicated at 500 W for 30 min. The mixtures were then filtered through a 0.22 μm syringe filter, and the filtrate was used for HPLC analysis. Drug loading (DL) and encapsulation efficiency (EE) were calculated as follows:

$$\text{DL}\%=\frac{\text{Weight of drug in micelles}}{\text{Weight of drug}-\text{loaded nanoparticles}}\times 100\%$$

$$\text{EE}\%=\frac{\text{Weight of drug in micelles}}{\text{Weight of feeding drug}}\times 100\%$$

### 3.5. In Vitro Dissolution Study

*In vitro* drug release studies were carried out in triplicate as follows [20]: SM-loaded nanoparticles (10.0 mg) were introduced into a dialysis bag (molecular weight cut-off 3.4 kD) and then placed in 100 mL of phosphate buffer solution (PBS, pH 7.4) containing 40% (*v*/*v*) PEG 400 [27] at 37 ± 0.5 °C with stirring. Samples (each of 2 mL) were withdrawn periodically, and then filtered through a 0.45 μm syringe filter. After each withdrawal, an equal volume of the dissolution medium was added to maintain a constant volume. Drug content was determined by the HPLC method described above.

### 3.6. Cytotoxicity Test

Human hepatoblastoma HepG2 cell lines were cultured in DMEM equilibrated with 90% humidified atmosphere of 5% CO_2_ in air at 37 °C. The medium was supplemented with 10% FCS and 200 mg/L SM solution. The Cell Counting Kit-8 (CCK-8) assay was used to measure cell cytotoxicity. HepG2 cells were seeded at a 3 × 10^4^ cells per well in 96-well plates (Costar^®^, Coring Inc., Coring, NY, USA) in medium and incubated for 24 h. The medium was then replaced with 100 μL of medium containing various equivalent concentrations of SM solutions or nanoparticles. The untreated cells were used as the control. The plates were incubated for another 24 h (or 48 h, 72 h), and cytotoxicity was measured using CCK-8 kits. The absorbance was measured at a test wavelength of 450 nm using a microplate reader (Bio-Rad Model 550, Segrate, Italy). The percentage of cell viability (CV %) was calculated based on the following equation:

$$\text{CV}\%=\frac{A_{\text{treated}}}{A_{\text{treated}}}\;\times \;100\%$$

where A_treat_ and A_control_ were the absorbance of the treated cells and the control, respectively. Experiments were carried out in six wells and tested three times.

### 3.7. Cell Uptake and Flow Cytometry Study

The capacity for cellular internalization of C6-loaded nanoparticles was visualized and quantified by microscope and fluorometry, respectively. HepG2 cells were seeded at a density of 2 × 10^5^ cells for each well in a 12-well plate (Costar^®^) and incubated for 24 h. Then, cells were replenished with serum-free medium containing free C6 or C6-loaded nanoparticles for 1 h. After incubation, cells were washed with ice-cooled PBS before they were fixed in 70% ethanol. Finally, the fixed cells were observed under a fluorescence microscope (BX51TF, Olympus, Tokyo, Japan).

To quantify the cellular uptake of the fluorescence probe C6, HepG2 cells were plated at a density of a 3 × 10^4^ cells per well in 12-well plates. The cells were incubated with free C6 or C6-hm-BSP nanoparticles in serum-free medium, washed 3 times with pH 7.4 PBS and then harvested by trypsinization. The intracellular fluorescence intensity was measured with a FACSCalibur flow cytometer (BD Biosciences, San Francisco, CA, USA). Approximately 1.0 × 10^4^ cells were counted to determine the trend of micelle uptake by the HepG2 cells.

### 3.8. Tissue Distribution Study

All animal procedures were conducted in accordance with the Guidelines for the Care and Use of Laboratory Animals and were approved by the Institutional Animal Care and Use Committee at Ningxia Medical University.

To understand and compare the biodistribution of free drug and drug-loaded hm-BSP nanoparticles, a lipophilic dye (DIR) that strongly absorbs the fluorescence in the near infra-redregion [28] was encapsulated into the hm-BSP nanoparticles. The DIR nanoparticles were prepared according to the above-mentioned method at a loading capacity of 0.089 μg/mL.

DIR-loaded nanoparticles were injected intravenously into mice *via* the tail vein. At 30 min after the injection, mice were imaged using a Kodak^®^ *in vivo* imaging systemFx Pro (Carestream Health Inc., Rochester, NY, USA). The images were acquired using epiiluminationat an excitation wavelength of 730 nm and an emission wavelength of 790 nm. Then, the mice were sacrificed and major organs were harvested for *ex vivo* imaging. The free DIR in PBS, which used Cremophor EL to solubilize at an equivalent concentration, was also injected into mice to compare the biodistribution of the DIR nanoparticles. Results were analyzed using Kodak^®^ imaging software (Carestream Health Inc.). All experiments were repeated in three different animals and representative pictures are shown.

### 3.9. Data Statistics

All of the results are expressed as the mean and standard deviation. The statistical analysis was performed by Student’s *t*-test using SPSS statistics software (SPSS software version 16.0, IBM, Armonk, NY, USA). The *p*-value < 0.05 was considered as statistically significant.

## 4. Conclusions

In the present study, SM was loaded into the nanoassembly of BSP conjugates with stearic acid and evaluated for the development of hepatic-targeted nanoparticles. The obtained nanoparticles appeared as spheres with an average size of 200 nm. SM was molecularly encapsulated into the nanoparticles at a loading efficiency of 78.9% and drug loading of 7.31%. The *in vitro* dissolution of the drug-loaded nanoparticles exhibited a typical sustained release profile. Compared to the drug solution, the developed nanoparticle formulation loaded with the drug improved cytotoxicity and cell uptake in HepG2 cell lines *in vitro*. These results suggest that hm-BSP derivatives are potentially effective nanocarriers for hepatic-targeted drug delivery, and the SM-hm-BSP nanoparticles will provide a better choice for enhanced clinical efficacy of SM preparations.

## Figures and Tables

**Table 1:** Physicochemical properties of SM-hm-BSP nanoparticles.

Sample	Diameter (nm)	PDI	Zeta (mV) ^a^	EE (%)	DL (%)
SM-hm-BSP	200.83 ± 8.10	0.25 ± 0.04	−0.36 ± 0.93	78.86 ± 0.66	7.31 ± 0.05

^a^ The zeta potential of micelles in distilled water at 1.50 mg/mL.
